# Bio-functionalized carbon dots for signaling immuno-reaction of carcinoembryonic antigen in an electrochemical biosensor for cancer biomarker detection

**DOI:** 10.1186/s11671-024-03980-3

**Published:** 2024-02-29

**Authors:** Amarnath Chellachamy Anbalagan, Jyoti Korram, Mukesh Doble, Shilpa N. Sawant

**Affiliations:** 1 Chemistry Division, Bhabha Atomic Research Centre, Trombay, Mumbai 400085 India; 2 Department of Cariology, Saveetha Dental College and Hospitals, Saveetha Institute of Medical and Technical Sciences, Chennai, 600077 India; 3 Homi Bhabha National Institute, Anushaktinagar, Mumbai 400094 India

**Keywords:** Carbon dots, Electrochemical biosensor, Cancer biomarker, Bio-conjugation, Polyaniline, Horseradish peroxidase

## Abstract

Early diagnosis of cancer demands sensitive and accurate detection of cancer biomarkers in blood. Carbon dots (CDs) bio-functionalization with antibodies, peptides or aptamers have played significant role in cancer diagnosis and targeted cancer therapy. Herein, a biosensor for detection of cancer biomarker carcinoembryonic antigen (CEA) in blood serum has been designed using CDs bio-functionalized with HRP-conjugated CEA antibody (CUCDs@^CEA^Ab2) as detection probe. CDs were synthesized by upscaling of cow urine, a nitrogen rich biomass waste, by hydrothermal method. Detection probe based on CDs resulted in 3.5 times higher sensitivity as compared to conventional electrochemical sandwich immunoassay. To further improve the sensor performance, hyper-branched polyethylenimine grafted poly amino aniline (PEI-*g*-PAANI) was used as the sensing interface, which enabled immobilization of higher amount of capture antibody. Detection of CEA in human blood serum coupled with wide linear range (0.5–50 ng/ml), good specificity, stability, reproducibility and low detection limit (10 pg/ml) signified the excellence of CUCDs based CEA immunosensor. CUCDs exhibited excitation wavelength dependent fluorescence property and showed strong blue emission under UV irradiation. MTT assay indicated that the material is not toxic towards human dental pulp stem cells (hDPSCs) and MG63 osteosarcoma cells (cell viability > 90%). The present study demonstrates a methodology for valorization of animal waste to a cost-effective carbon based functional nanomaterial for clinical detection of cancer biomarkers.

**Supplementary Information:**

The online version contains supplementary material available at 10.1186/s11671-024-03980-3.

## Introduction

Recently cancer surpasses heart diseases as the leading cause of death worldwide [1]. Detection of cancer at the earliest stage supports the treatment significantly and increases the chance of survival of patient. Biosensors hold enormous potential for detection of critical cancer biomarkers and for determining effectiveness of anti-cancer drug during treatment. However, detection of biomarkers at low concentrations has always been a challenging task in clinical diagnosis. Hence, the development of highly sensitive and reliable detection techniques is essential for quantification of cancer biomarkers [2]. Several methods are extensively used for the detection of various cancer biomarkers, which include optical, electrochemical, piezoelectric and calorimetric based biosensors [3]. Among these, electrochemical technique affords cost-effective, portable, rapid and sensitive way of detection. In any ultra-sensitive electrochemical detection event, the role of detection probe and immobilization matrix (IM) is of utmost importance, which widens the linear range and enhance the output signal several folds [4]. IM should contain a large amount of functional groups and ability to transduce biological signal to electrochemical signal. To address this, functional group enriched conducting polymers based IM are desirable. In the case of detection probes, several nanomaterials like metal nanoparticles, metal oxides, inorganic complex, carbon nanotubes, carbon dots, have been extensively explored [5]. In our previous reports, amine and carboxylic acid group enriched PANI based IMs were developed by making composite with bovine serum albumin (BSA) and silver nanoparticles (Ag NPs) [6], and thiol-ene click chemistry respectively [7, 8]. These sensing surfaces were used as generic IMs for detection of metabolites [7], single [6, 7] and multiple cancer biomarkers [8].

Among nanomaterials, carbon-dots (CDs) have received remarkable interest and are extensively explored in the area of energy and biomedical applications mainly due to their small size, abundant functional groups, biocompatiblity, photoluminescence and electro-catalytic property [9]. CDs are nano-sized carbon based materials with considerable amount of oxygen and hydrogen. Their size and easy bio-conjugation ability has made CDs as a promising candidate for electrochemical biosensing application [10–12]. Syntheses of CDs have been widely classified in two categories viz. top-down and bottom-up approach. Former approach is associated with the cleavage of macroscopic carbon precursors in to smaller CDs while later approach is associated with the carbonization [13–17].

Chemicals, natural materials and biomass have been used as the main precursors for the CDs synthesis. Biomass is a good choice as precursor since it is renewable, cost-effective, eco-friendly, easily and abundantly available. Agriculture related sources like grass, leaves, fruit peels, tea extract, and coconut husk have been used by several researchers for CDs synthesis [18–20] and used for bioimaging application [19, 21]. Cow urine is one of the major biomass produced from agriculture sector. Cow urine is composed of 95% water, 2.5% urea as well as minerals, salts and hormones [22]. While many researchers studied the antimicrobial and medicinal property of cow urine, its use for synthesis of nanomaterials is not reported. Total nitrogen in the cow urine is ranged from 6.8 to 21.6 g N l^−1^, of which an average of 69% is present as urea. Rest of the nitrogen is contributed from allantoin, hippuric acid, creatinine, creatine, uric acid, xanthine plus hypoxanthine, free amino acid N and ammonia [23]. Due to presence of significant amount of nitrogen containing compounds, cow urine can be used as the excellent precursor for synthesis of nitrogen doped CDs.

In the present work, cow urine derived carbon dots (CUCDs) was synthesized by hydrothermal treatment of cow urine, bio-functionalized with enzyme conjugated antibody and used as detection probe to improve the sensitivity of biosensor for CEA detection. CUCDs were characterized by Fourier transform infrared (FTIR), UV–Visible, fluorescence spectroscopy, and TEM studies. Furthermore, hyper-branched polyethylenime grafted poly amino aniline (PEI-*g*-PAANI) based amine enriched immobilization matrix was used to fabricate electrochemical biosensor. Present work demonstrates simple approach of converting cow urine into biocompatible, fluorescent carbon dots which were further exploited to construct an efficient detection probe in sandwich assay based electrochemical biosensor for cancer biomarker detection.

## Experimental

### Materials

*O*-phenylenediamine (*o*-PDA), hydroquinone, polyethylenimine (PEI), hydrogen peroxide, ammonium persulfate, di-sodiumhydrogen phosphate, mono-sodiumdihydrogen phosphate, glutaraldehyde and bovine serum albumin were procured from Sigma Aldrich (Merck). monoclonal CEA antibody (^CEA^Ab1), horseradish peroxidase conjugated CEA antibody (^CEA^Ab2), purified CEA antigen was purchased from Bioss USA. Zensor R&D screen printed carbon electrode (SPE) used for all electrochemical characterization. Local breed cow urine was used as the source to synthesize carbon dots.

### Characterization

PEI-*g*-PAANI and CUCDs were characterized by various spectroscopic techniques and transmission electron microscopy (TEM). ATR-FTIR spectroscopy of PEI-*g*-PAANI and CUCDs based samples were performed using Bruker compact FTIR-Spectrometer (ALPHA-II). Fluorescence (FL) and UV–visible spectra were recorded using JASCO FP-8500 spectrofluorometer and JASCO-UV-650 spectrophotometer, respectively. TEM images were recorded from Thermo Scientific 300 G3 microscope (accelerating voltage 300 kV). The electrochemical investigations like cyclic voltammetry and chronoamperometry were carried out using Metrohm Autolab electrochemical work station. Electrochemical detection was carried out in phosphate buffer solution (PBS) of pH 7.4 with hydroquinone (HQ) and H_2_O_2_. EIS was performed in 0.1 M KCl containing 1:1 mixture of 5 mM K_3_[Fe(CN)_6_] and K_4_[Fe(CN)_6_] (frequency 1 MHz to 1 Hz, amplitude 10 mV, at 0.4 V). All the antibody conjugation and binding/sensing experiments were performed in PBS (pH 7.4), which is ideal medium to process biomolecules as cited in several related work [24, 25]. All the experiments were performed at ambient temperature (25 °C) [26].

### Synthesis of CUCDs

20 ml of filtered cow urine sample was heated in a hydrothermal set-up at 200 °C for 5 h. After natural cooling, brown coloured viscous mixture was dialyzed for 12 h under constant stirring. Dialyzed sample (50 ml) was evaporated to 5 ml, mixed with equal volume of ethanol to precipitate inorganic salts from CUCDs which was removed by centrifugation (10,000 rpm, 10 min). Ethanol present in the supernatant was evaporated to get CUCDs in aqueous solution.

### Bio-conjugation of CUCDs with HRP conjugated CEA antibody (CUCDs-^CEA^Ab2)

1 ml of CUCDs was treated with 1:1 EDC (0.1 M)/NHS (0.05 M) mixture, 5 μg/ml HRP conjugated CEA antibody (^CEA^Ab2) and kept at 4 °C for 24 h. Thus obtained mixture was dialyzed to remove unreacted contents and ^CEA^Ab2 bio-conjugated CUCDs (CUCDs@^CEA^Ab2) was obtained. This was used as the detection probe in sandwich electrochemical immunoassay for CEA detection.

### Synthesis of PEI-*g*-PAANI

PEI-*g*-PAANI was synthesized in two-steps. In the first step, 1 M HCl containing 0.1 M *o*-PDA was electro-polymerized [27] on SPE using cyclic voltammetry from − 0.3 to 1 V at 50 mV/s for 10 cycles to get poly amino aniline (PAANI). In the second step, thus prepared PAANI modified SPE was rinsed with deionized water and treated with 1% glutaraldehyde followed by 1 mg/ml of PEI solution to get PEI-*g*-PAANI modified SPE and used for all studies. PEI-*g*-PAANI was also synthesized by a conventional oxidative chemical polymerization method to compare the chemical nature. In brief, solution containing 1.081 g o-PDA in 50 ml of 1 M HCl was prepared. To this, 50 ml of APS solution (4.56 g APS in 1 M HCl) was added dropwise for 10 min under constant stirring. The reaction was carried out for 24 h at room temperature to yield green coloured PAANI salt, which was filtered using Whatmann 41 filter paper. The precipitate was washed with 1 M HCl, deionized water and PBS pH 7.4. Thus filtered PAANI was treated with 1% glutaraldehyde followed by 1 mg/ml PEI solution to get PEI-*g*-PAANI. The FTIR spectra of PEI-*g*-PAANI synthesized by chemical and electrochemical pathway are shown in Fig. S4.

### Construction of CEA sandwich biosensor

In the first step, PAANI-g-PEI deposited SPE was modified with monoclonal CEA antibody (^CEA^Ab1) (500 ng/ml, incubation time 30 min at room temperature) via glutaraldehyde coupling (1% solution, incubation time 10 min at room temperature) followed by blocking of –CHO active sites by 1 wt% BSA solution (incubation time 10 min) to avoid non-specific binding. After each treatment, the electrode was rinsed with PBS by placing the electrode in a beaker containing 5 ml PBS for 30 s. This process was repeated twice with new phosphate buffer solution. Known and different concentrations of CEA were incubated with thus prepared electrode for 30 min. Then detection probe CUCDs@^CEA^Ab2 (10 μl) was incubated on the CEA bound electrode with for 60 min. After rinsing the electrode with PBS, chronoamperometry (CA) was carried out in PBS containing HQ (3 mM) and H_2_O_2_ (3 mM). The amperometric current response obtained on reduction of H_2_O_2_ by CUCDs@^CEA^Ab2 bound electrode measured. CA measurements were carried out at 0 V for 10 s and − 0.1 V for 10 s as the reduction of H_2_O_2_ by HRP present in CUCDs@^CEA^Ab2 occurred at this potential in presence of HQ. For a particular CEA concentration, the difference in value of current (ΔI) for 3 mM H_2_O_2_ and 0 mM H_2_O_2_ ($\Delta {\text{I}} = {\text{I}}_{{{\text{H}}_{{2}} {\text{O}}_{{2}} }} {-}{\text{I}}_{{{\text{blank}}}}$) was used to obtain a calibration plot of ΔI vs. concentration of CEA. For real sample analysis, 10 μl of human blood serum was added on the ^CEA^Ab1 modified SPE-PEI-*g*-PAANI electrode instead of CEA standard solution. The detection limit of proposed biosensor was determined using the formula 3σ/S, where σ is the standard deviation of the blank signal and S is the slope of the linear calibration plot. For interference study, 10 µl of 5 mM of ascorbic acid, uric acid, l-cysteine, l-arginine, BSA (1 mg/ml), FBS (1 mg/ml), AFP (50 ng/ml) were used in each sensor electrode instead of CEA antigen.

### Real sample preparation

Blood sample collected in vial was kept undisturbed for 0.5 h for coagulation and coagulated part was separated by centrifuge (5000 rpm, 8 min). The supernatant was stored at − 20 °C for further electrochemical analysis and chemiluminescent microparticle immunoassay (CMIA).

### Viability of human dental pulp stem cells (hDPSCs) and MG63 osteosarcoma cells in the presence of synthesised carbon dots

The % cell viability with respect to different concentrations (12.5, 25, 50, 100 and 250 µg/ml) of CUCDs from the stock concentration of 1 mg/ml was carried out by MTT assay using hDPSCs and MG63 cells [20, 28]. The individual cells were grown separately and 1 × 10^4^ cells added to the each well of 96 well plate and grown using Dulbecco's Modified Eagle Medium (DMEM) F12 and DMEM medium supplemented with 10% FBS with 1% antibiotic–antimycotic mix, respectively. The different concentration of CUCDs prepared using respective medium were incubated for 24 and 72 h. Subsequently, after the predetermined time interval, MTT reagent was added to the respective well and incubated further about 3 h and added 200 µl DMSO to dissolve the formazan crystals and produced purple colour which was measured using spectrophotometer (absorbance read at 570 nm) using BioTek Synergy H1 modular multimode microplate reader, Agilent, USA.

$$\left( \% \right) \;Cell\;viability = \frac{Sample\;OD}{{Control\; OD}} \times 100$$

### Statistical analysis

One way ANOVA was performed with Microsoft excel software to ascertain the differences in the cell viability in the MTT assay. A *p* value less than 0.05 is considered as significant.

## Results and discussion

### CUCDs

The hydrothermal treatment of cow urine at 200 °C for 5 h followed by dialysis afforded CUCDs. Cow urine contains 95% water and rest is composed of minerals, salts, urea, enzymes, and amino acids. Even though it is easily available, abundant, non-toxic, renewable, converting this type of bio-waste in to useful product is always desirable.

Zhuang et al. used human urine to produce graphitic carbon nitride and used as the fluorescent probes for multi-color bioimaging of cells [29]. Xavier et al. synthesized fruit extract and human urine derived carbon dots and used for the detection of Hg^2+^ and cysteine using turn-off–on fluorescence technique [30]. In most of the work, CDs were employed for fluorescence-based detection due to its size and optical properties. However, CDs are seldom used as probe to enhance to electrochemical signal in sandwich biosensor for the detection of cancer biomarkers.

**Fig. 1:**
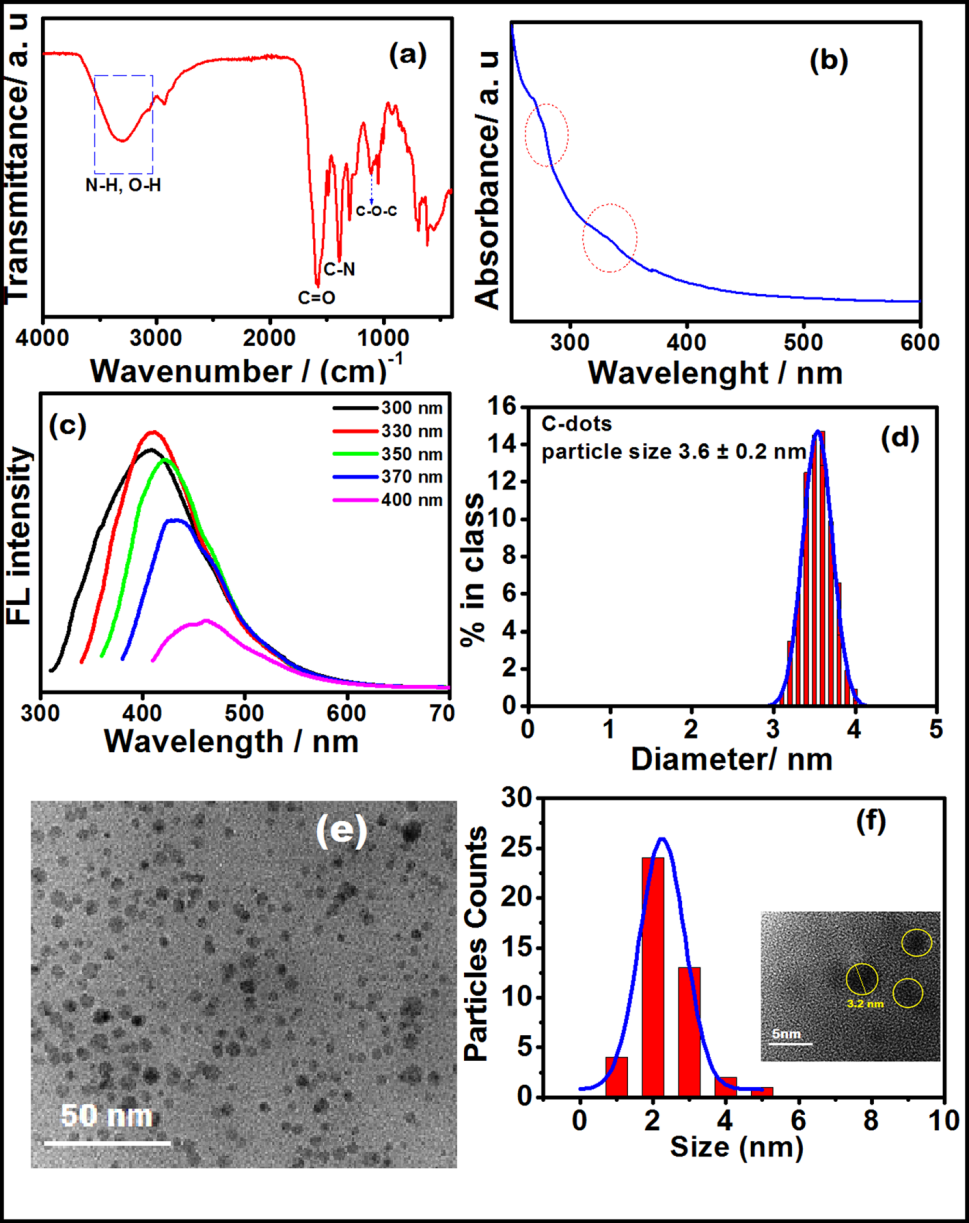
**a** FTIR, **b** UV–visible, **c** Fluorescence spectra, **d** DLS, **e** TEM and **f** particle size distribution of CUCDs (inset HR-TEM image of CUCDs)

FTIR spectrum of CUCDs (Fig. 1a) displayed the characteristic peaks at 3340 cm^−1^ (stretching vibrations of O–H and N–H), 2910 and 2870 cm^−1^ (stretching vibration of aliphatic C–H), a broad and intense peak from 1710 to 1502 cm^−1^ (merging of C=O, bending vibration of N–H, C=C) and 1300 cm^−1^ (CN groups). The bands at 3300 and 1615 cm^−1^ corresponded to N–H bending vibration of amino group confirms the presence of amino group in the CUCDs [31]. This is due to the carbonization of nitrogen containing compounds like urea, ammonia, allatonin, creatinine, and ceratine present in the cow urine.

The UV absorption spectrum (Fig. 1b) did not show significant peaks, however, humps were observed at 271 and 371 nm corresponding to π–π* transition of C=C bonds in the graphitized carbon and n–π* transition of the C=O and C=N group, respectively. CUCDs displayed blue fluorescence under excitation at 300–400 nm wavelength. At the excitation wavelength of 330 nm, maximum emission was obtained at 410 nm. As the excitation wavelength increased, the maximum emission wavelength was red-shifted, indicating excitation-wavelength dependent emission characteristics (Fig. 1c). This behaviour is due to the different sizes and surface defects of the CUCDs, as observed for most light-emitting CDs and graphene quantum dots [30].

Quantum yield is important parameter for the CDs, which describes the ability of particle to release absorbed light as photons. The quantum yield for biomass derived CDs reported in literature is mostly < 10% as summarized in Table S1. However, doping or surface functionalization can enhance the quantum yield of CDs. In the present work, the values of QY obtained for the CUCDs is 5.69% (Fig. S1) which is better than CDs prepared using biomass like bagasse, soy milk and cucumber juice (Table S1). Most of biomass derived CDs were used as bio-imaging probes in which QY is very important. CDs as a nanocarrier for electrochemical detection of cancer biomarker is seldom reported. To use as a nanocarrier, size and surface functional groups are also important in addition to the QY. In these aspects, CUCDs is a suitable candidate to accommodate antibodies, and was hence utilized for sandwich electrochemical biosensor. In the present work, CDs are used as the nano-carrier to accommodate HRP conjugated PSA antibody. With increase in size of CDs, more amount of antibody can be loaded over the probe, however, there will be a red shift in the emission spectra [24].

Particle size was determined by DLS (Fig. 1d), which showed 3.6 nm average hydrodynamic diameter. Consequently, size was also studied by TEM, where CUCDs showed discrete spherical morphology (Fig. 1e) with average particle size of 3.2 nm (Fig. 1f) revealing the well-dispersed nature of CUCDs in aqueous solution. Reproducibility is one of the general issues with the biomass derived nanomaterials. However, using biomass waste as starting material has several advantages including valorization of waste into value added products. To evaluate the reproducibility of CUCDs synthesis method, a set of CDs has been synthesized using urine samples from different cows and urine samples collected from same cow at different time of the day. The results are summarized in ESI Fig. S2.

These results signify the presence of amine and carboxylic acid functional groups on the surface of the CUCDs. Due to its size and functional groups, it can be efficiently conjugated to biomolecules and was thus used to develop probe to signal the sandwich immunosensor for cancer biomarker detection.

### PEI-*g*-PAANI: an amine enriched immobilization matrix

Conventionally, PANI with other nanomaterials like metal NPs, biopolymer, carbon nanotubes or graphene are used as the matrix in a biosensor. Designing PANI with dense functional group can help to immobilize more amounts biomolecules like enzymes or antibodies leading to biosensor with improved analytical performance [32–38]. In the present work poly amino aniline was covalently modified with hyper-branched polyethylenimine via simple glutaraldehyde coupling chemistry to get PEI-*g*-PAANI. Initially poly amino aniline (PAANI), was synthesized by electro-polymerization of *o*-phenylene diamine (*o*-PDA) on screen printed carbon electrode (SPE) using cyclic voltammetry in 1 M HCl solution at 50 mV/s for 10 cycles. Significant increase in redox current during consecutive potential cycling indicates the deposition of PAANI films on SPE (Fig. S3). Thus, synthesized PAAN modified SPE was washed with water and treated with glutaraldehyde followed by the PEI to get PEI-*g*-PAANI modified SPE.

Chemical nature of PAANI and PEI-*g*-PAANI was studied by FTIR spectroscopy. The FT-IR spectrum of both the samples (Fig. S4) showed the signature peaks for conventional PANI at 1510, 1605, 1310 and 805 cm^−1^ attributed to C=C stretching of benzenoid ring (B), C=C stretching of quinonoid ring (Q), C–N stretching of the secondary aromatic amine and C–H out-of-plane bending of 1,4-disubstituted ring respectively [39]. However, PEI-*g*-PAANI displayed an additional peak at 3220 cm^−1^ indicating the presence of free amino group in the polymer chain (Fig. S4b). This confirms the grafting of hyper branched PEI on the polymer chains [40].

### Electrochemistry of PAANI-g-PEI

**Fig. 2:**
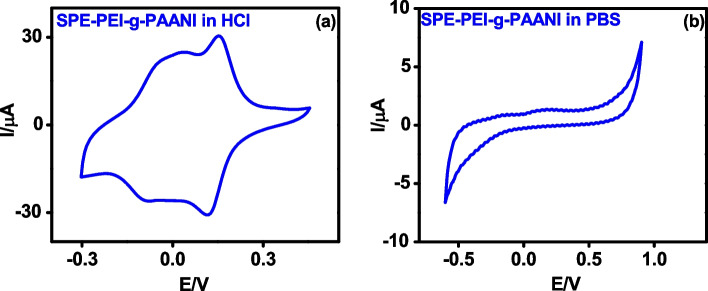
CV of PEI-*g*-PAANI in **a** 1 M HCl and **b** PBS pH 7.4 at scan rate 50 mV/s

Cyclic voltammetry was carried out for PAANI-*g*-PEI in acidic medium and PBS (pH 7.4). In the acidic medium, CV curves of PAANI-*g*-PEI displayed two redox peaks similar to conventional PANI in which first one is attributed to leucoemeraldine-emeraldine transition and second one is due to emeraldine-pernigraniline transition (Fig. 2a) [41]. However, in PBS (pH 7.4) no redox peak was observed as in the case of conventional PANI (Fig. 2b) [42].

### Immobilization efficiency of PEI-*g*-PAANI

**Fig. 3:**
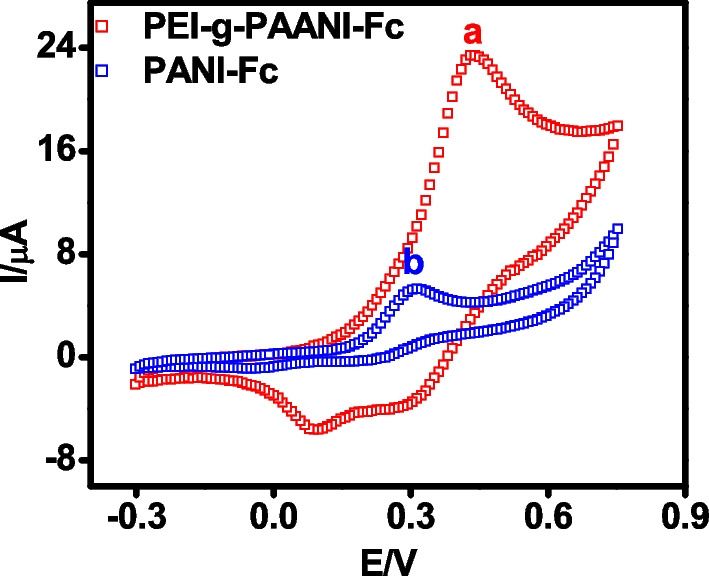
CV of ferrocene grafted **a** PEI-*g*-PAANI and **b** conventional PANI on SPE in PBS at 50 mV/s

Immobilization efficiency was investigated by conducting cyclic voltammetry and electrochemical impedance spectroscopy. Initially, an attempt was made to quantify the surface concentration of free amino group (–NH_2_) on the sensing platform, which gives an estimate of the immobilization capacity of the PEI-*g*-PAANI as compared to conventional PANI. Generally, grafted redox species on the surface of the electrode can be quantified using the formula

1 $${\text{Q}}/{\text{nFA}}$$

where Q is anodic charge developed during cyclic voltammetric scan of modified electrode, n is the number of electrons involved in the redox reaction, F is the Faraday constant and *A* is the surface area of the working electrode [43]. However, –NH_2_ groups in PEI-*g*-PAANI modified electrode is not redox active and hence, quantifying through electrochemical method is difficult. Therefore, the electrode was incubated with ferrocene carboxaldehyde (Fc-CHO) to make the electrode surface redox active. The –CHO of Fc-CHO reacted with –NH_2_ group of modified electrode to form ferrocene grafted PEI-*g*-PAANI via oxime bond. CV curve of Ferrocene grafted PEI-*g*-PAANI modified electrode (Fig. 3) displayed redox peak with 3.2 times higher current than ferrocene grafted PANI modified electrode indicating the presence of more number of –NH_2_ group on its surface. Using the Eq. 1, the surface concentration of redox species on the modified electrode was found to be 36 nmol cm^−2^ which is 3.3 times higher than that of ferrocene grafted conventional PANI based electrode (11 nmol cm^−2^). This finding authenticates the presence of higher amount of active sites for immobilization, making PEI-*g*-PAANI modified electrode as the suitable matrix for sensing application.

Immobilization efficiency of PEI-*g*-PAANI was further studied by cyclic voltammetry and EIS measurements. CV of PEI-*g*-PAANI and bio-conjugated PEI-*g*-PAANI was recorded in 0.1 M KCl with 5 mM K_3_[Fe(CN)_6_] and K_4_[Fe(CN)_6_]. Redox peak current of bio-conjugated PEI-*g*-PAANI decreased significantly after bio-conjugation with ^CEA^Ab1 (Fig. S5a). This could be due to the blocking of electron transfer by conjugated biomolecules on PEI-*g*-PAANI surface [44]. To further investigate the immobilization efficiency, EIS of PEI-*g*-PAANI before and after bio-conjugation was carried out and compared with conventional PANI. The Nyquist plot (Fig. S5b and c) of both the electrodes, before and after bio-conjugation displayed an arc (at higher frequency region) followed by a line (at lower frequency region). After immobilization step, the diameter of arc i.e., charge transfer resistance (R_CT_) increased significantly. This increase in R_CT_ (Table S2) is significantly high (250 Ω) in the case of PEI-*g*-PAANI (Fig. S5b) vis-à-vis resistance of 20 Ω for conventional PANI (Fig. S5c), signifying the binding of higher concentration of antibody on electrode of PEI-*g*-PAANI [7].

### Sandwich electrochemical immunosensor CEA detection

**Scheme 1.**
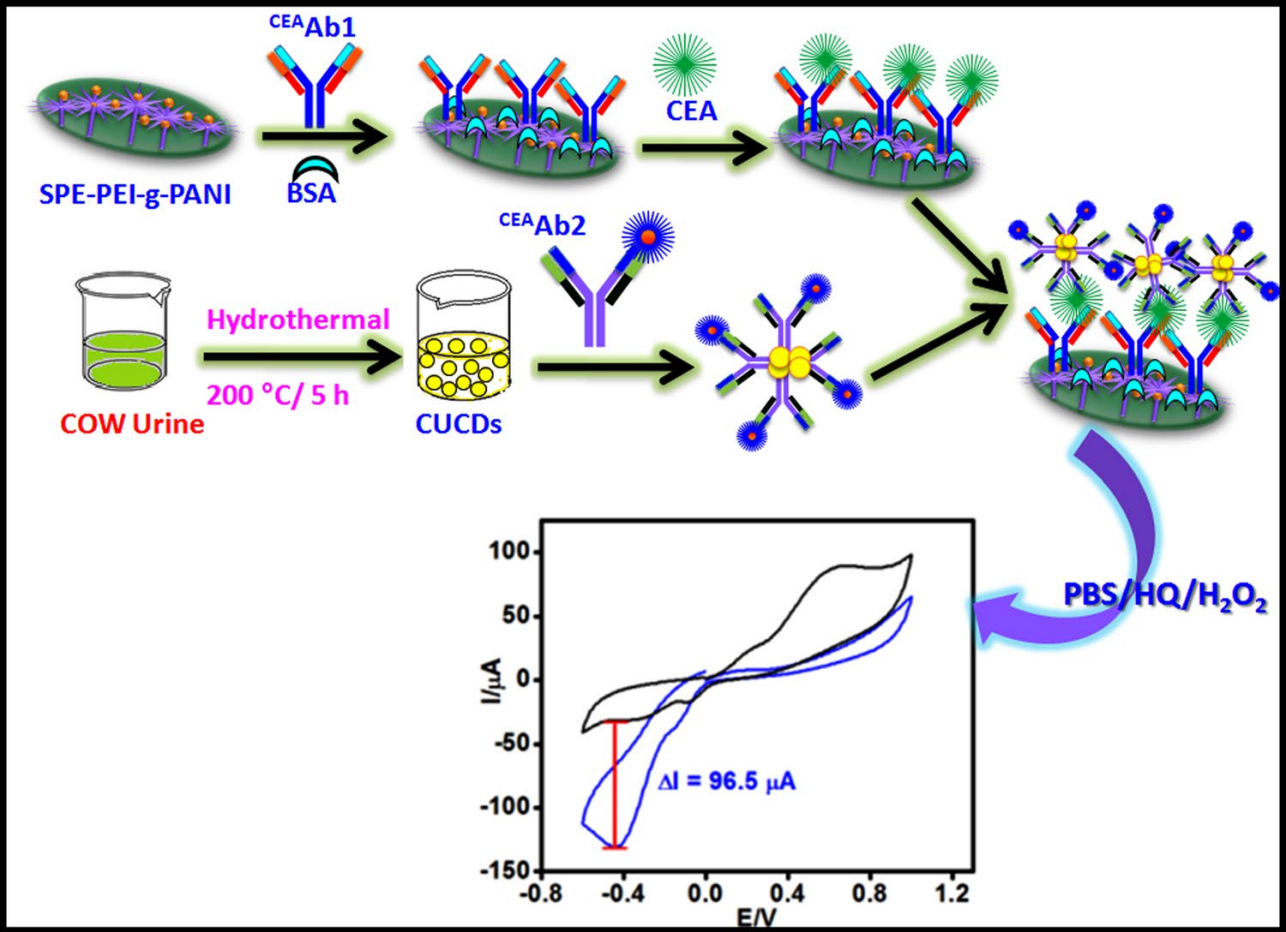
Construction of sandwich immunosensor for CEA detection and signal amplification

Carcinoembryonic antigen (CEA) is a serum based cancer biomarker that is elevated in the case of colorectal cancer. The average CEA level in serum of healthy adult is below 5 ng/ml, while CEA level above 20 ng/ml indicates the incidence of cancer [45]. Over-expression of CEA is generally associated with colorectal, breast, lung, ovarian and thyroid cancer. Hence detection of CEA has important role in clinical and biomedical applications. A sandwich electrochemical immunoassay for sensing of CEA was demonstrated using PEI-*g*-PAANI as immobilization platform and HRP conjugate CEA antibody (Ab2) functionalized CUCDs (CUCDs@^CEA^Ab2) as the detection probe for the signal amplification (Scheme 1). The three-step sandwich electrochemical immunoassay leads to increase in current response with increasing antigen concentration whereas in the case of direct single-step immunoassay, the current response decays due to passivation of the electrode surface.

### Optimization of experimental conditions

**Fig. 4:**
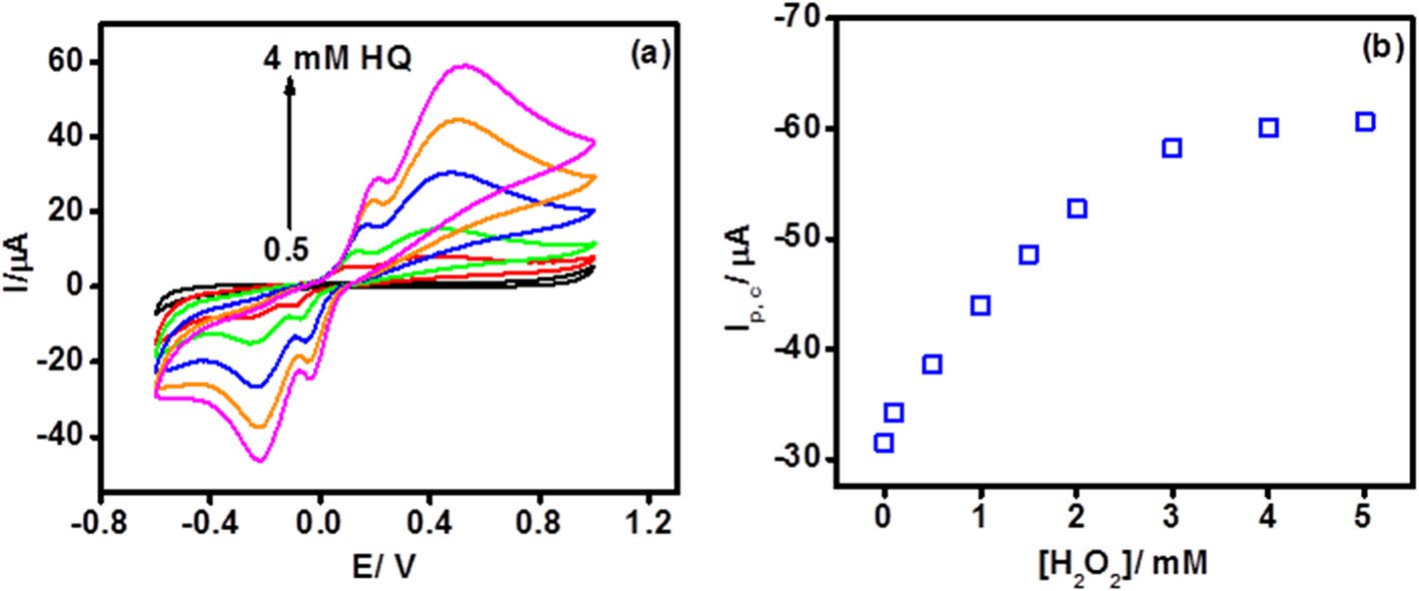
CV of **a** PEI-*g*-PAANI modified SPE in PBS with increasing concentration of HQ at 50 mV/s and **b** CV response of CUCDs@^CEA^Ab2 modified SPE-PEI-*g*-PAANI with increasing concentrations of H_2_O_2_ (at 50 mV/s) in PBS containing HQ (3 mM)

To accomplish ideal performance, analytical conditions like concentration of HQ and H_2_O_2_ is to be optimized. Figure 4a shows the CV of PEI-*g*-PAANI modified SPE in PBS containing increasing concentrations of redox mediator HQ. A distinct redox peak was witnessed when 3 mM HQ was employed and therefore 3 mM concertation of HQ was used in all experiments. To optimize, H_2_O_2_ concentration, CUCDs@^CEA^Ab2 was directly immobilized on PEI-*g*-PAANI modified SPE via glutaraldehyde coupling and CV was carried out with sequential addition of H_2_O_2_ in presence of HQ (Fig. 4b). At 3 mM H_2_O_2_, saturation in response current was observed and thus 3 mM of H_2_O_2_ was used in all electrochemical experiments. Incubation time followed for ^CEA^Ab1, CEA antigen and CUCDs@^CEA^Ab2 binding during sensor construction was 30 min [7].

### Signal amplification by CUCDs

**Scheme 2.**
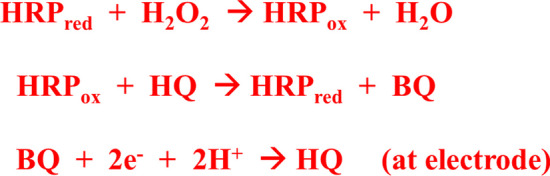
Mechanism of HRP catalyzed H_2_O_2_ reduction in presence of HQ

**Fig. 5:**
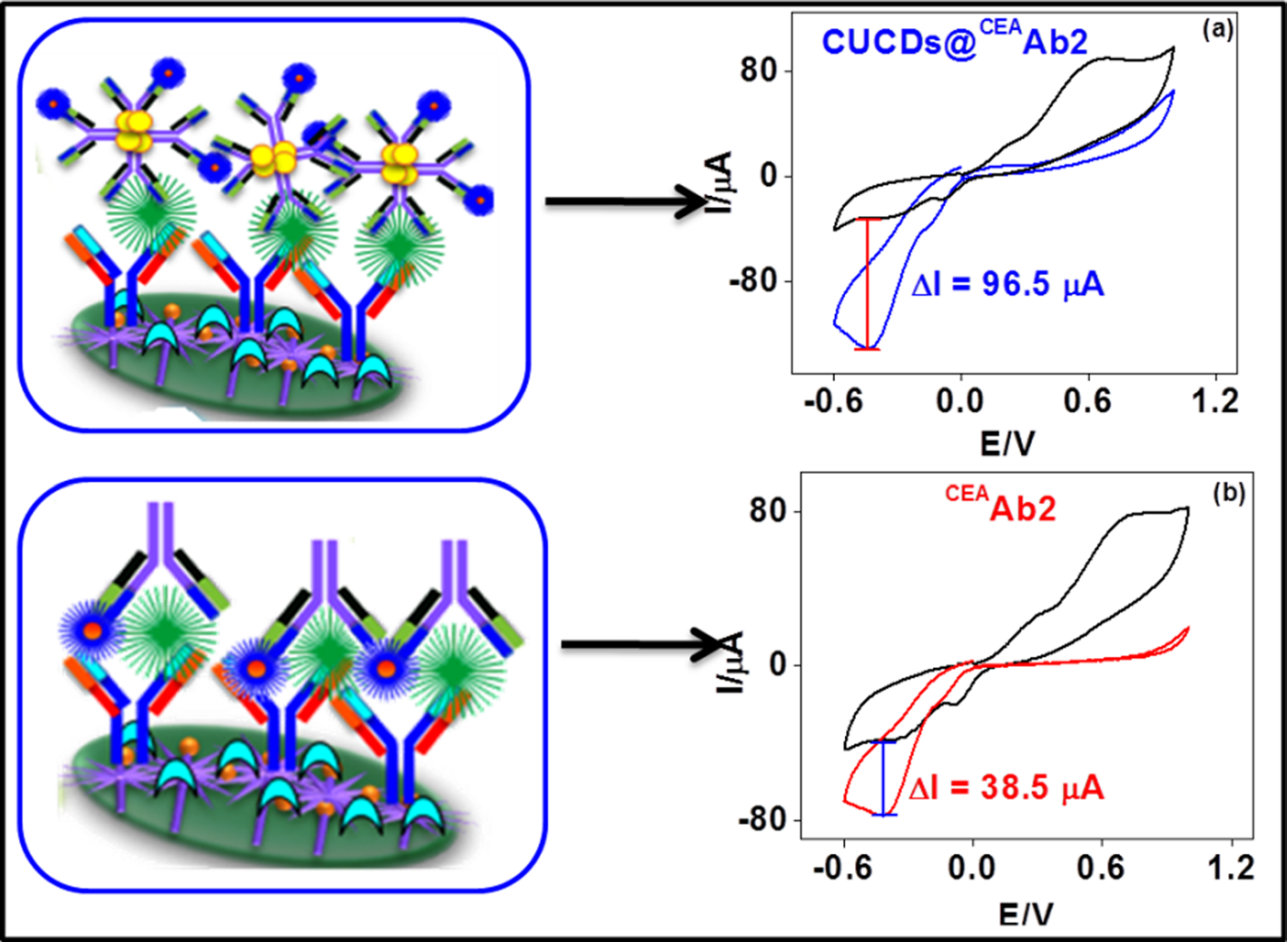
Cyclic voltammetry of sandwich immunosensor **a** with and **b** without CUCDs nano-carrier in PBS containing HQ in absence and presence of H_2_O_2_

Effect of antibody on CUCDs was studied by fluorescence spectral analysis. Fluorescence peak intensity (Fig. S6) of CUCDs decreased after treatment with antibody which signifies the effective bio-functionalization of CUCDs [46]. CUCDs was used as the carrier of HRP conjugated CEA antibody to signal the immuno-reaction. Generally HRP is used as the enzyme based tag along with the antibody in the sandwich immunoassay [47]. HRP enzyme catalyzes reduction of H_2_O_2_ in to H_2_O in presence of electron mediator like HQ, which gets oxidized to benzoquinone (BQ). BQ is reduced at the electrode surface to regenerate HQ (Scheme 2). This reaction produces redox signal during the binding of HRP tagged detection probe with antigen bound electrode surface [6, 7, 47]. In the present system, CUCDs was bio-functionalized by HRP conjugated CEA antibody (CUCDs@^CEA^Ab2) and used as the detection probe in sandwich immunosensor for CEA detection. During the detection event of CEA, the sensor with CUCDs@^CEA^Ab2 detection probe afforded 2.5 times higher redox current (Fig. 5a) as compared to ^CEA^Ab2 (Fig. 5b).

### Analytical performance of CEA immunosensor

**Fig. 6:**
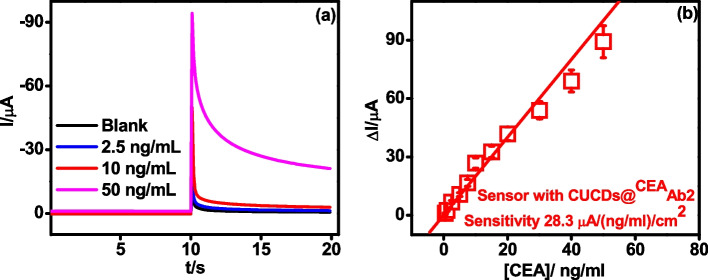
**a** Amperometric curves of CEA immunosensor with CUCDs@^CEA^Ab_2_ detection probe in 3 mM HQ and 3 mM H_2_O_2_ with different concentrations of CEA **b** calibration plot for CUCDs@^CEA^Ab_2_ based CEA immunosensor

Under optimal conditions, the electro-catalytic currents of the CUCDs@^CEA^Ab2 bound sensor increased with increase in concentration of CEA antigen (Fig. 6a). This increase of amperometric current was proportional to the CEA concentration in the range of 0.5 – 50 ng/ml (Fig. 6b and Fig. S7).

**Fig. 7:**
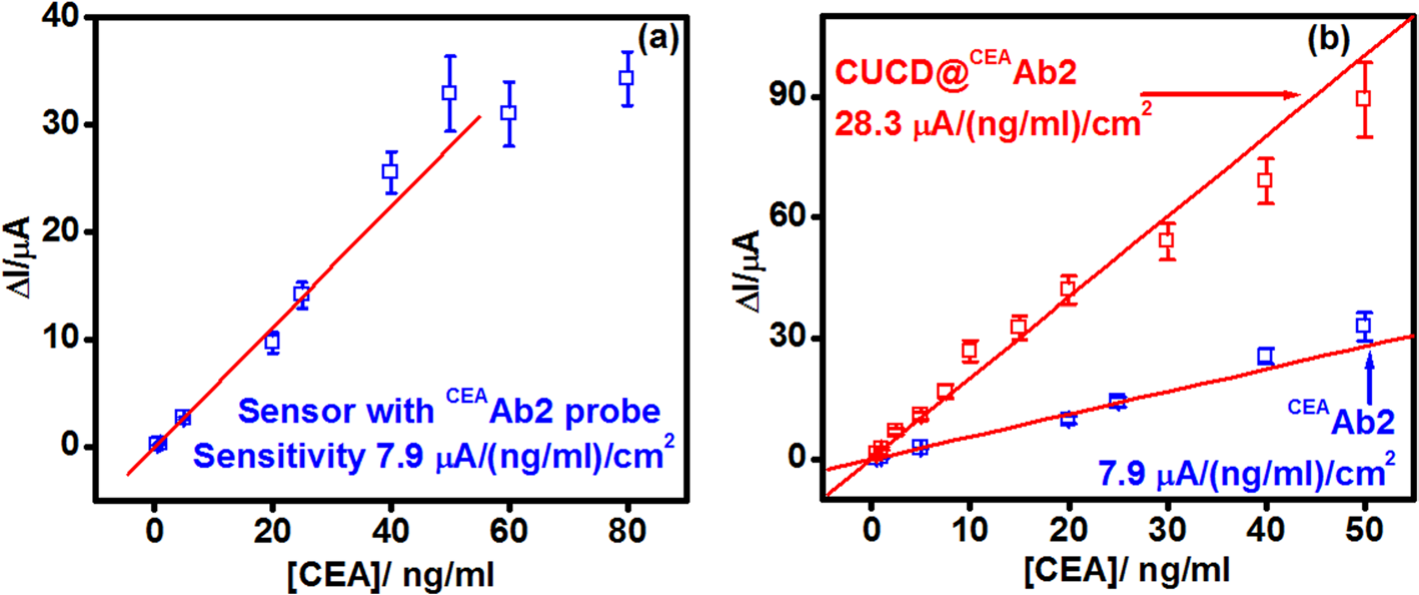
Calibration plot for **a** ^CEA^Ab2 and **b** its comparison with CUCDs@^CEA^Ab2 based CEA immunosensor

The sensor demonstrates dual amplification strategy via (1) immobilization matrix and (2) detection probe. PEI-*g*-PAANI matrix could immobilize a large amount of antibodies which can improve the linear range of biosensor. Consequently, nano-carrier present in the detection probe can accommodate more number of HRP conjugated antibody and which increases the resultant signal during the immuno-reaction between detection probe and antigen [5, 48–51]. Present method employed a single material in the sensing platform which can play key role in decreasing the fabrication and diagnosis cost. Linear range obtained is 5 times wider than the linear range obtained for electro-polymerized *o*-phenylenediamine over Au electrodes for CA 15–3 sensing [52] which emphasize the grafting of PEI on the PAANI. This evidenced that the enhancement in sensor performance is due to the grafting of PEI to the polymer chains. The sensor with CUCDs based detection probe afforded about 3.5 times higher sensitivity (28.3 μA/(ng/ml)/cm^2^) than the sensor conventional detection probe (7.9 μA/(ng/ml)/cm^2^) (Fig. 7a and Fig. S8) as seen from the Fig. 7b. This also further proves the efficient loading of ^CEA^Ab2 over CUCDs surface as demonstrated in Fig. 5a.

Hence, sensor provided wide linear range and low detection limit (10 pg/ml) for CEA detection (S/N ratio 3) which is adequate for detecting CEA in real human blood samples. Sensor fabricated using other sensing matrix are provided in the Table S3 of ESI for comparison. The performance of proposed sensor is better (Table S3) than the CEA biosensor fabricated using other conducting polymer based composites like polypyrrole (PPY) nanocomposite [33 pg/ml], Poly(3,4-ethylenedioxythiophene) (PEDOT) /PPY films [80 pg/ml] and graphene-PEDOT [450 pg/ml] in terms of limit of detection (LOD). Wang et al. utilized lecitin based tracing probe and HRP label for identifying CEA over Au electrode with a LOD of 3.4 ng/ml. Zhao et al. designed sensing matrix composed of graphene oxide, Au NPs, poly(indole-6-carboxylic acid) for label-free detection of CEA with LOD 20 pg/ml. As compared to multi-component probe and sensing matrix utilized in the above reports, the proposed biosensor provided better analytical performance is terms of LOD due to CUCDs probe designed to accommodate higher amount of detection antibody. Gu et al. demonstrated highly reproducible CEA biosensor with LOD of 10 ng/ml using thiol containing ferrocene molecules modified Au NPs as detection probe. Covalent bond between ferrocene molecule and Au NPs afforded stable electrochemical signal amplification during detection. Yang et al. designed 3D Au NPs-prussian blue-PEDOT based redox sensing matrix for the label-free detection of CEA with LOD of 10 ng/ml. As compared to these report, the proposed biosensor adopted simple synthesis strategy and cost-effective precursors to afford comparable LOD values. There are few reports which used nano-hybrid based detection probes (Table S3) where higher sensitivity is reported. For instance, highly sensitive CEA biosensor with very low LOD (17 fg/ml) has been demonstrated over Au NPS modified GCE and using Cu_2_O@Cu-MOF@Au as detection probe (Table S3). Designing a detection probe or sensing matrix with such combinations of materials requires tedious reaction procedure and conditions. For excellent sensor performance, percentage of individual entities and its spatial organization also needs to be controlled during synthesis and sensor fabrication [53]. The present methodology afforded simplicity in material synthesis, easy sensor construction than other sensor reported in literature.

### Specificity, reproducibility and stability of CEA immunosensor

**Fig. 8:**
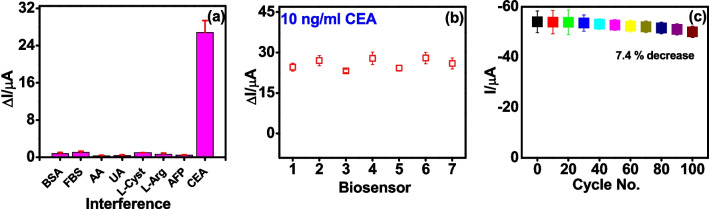
**a** Specificity studies of the biosensor in BSA, FBS, AA, UA, l-Cysteine, l-Arginine, AFP and CEA, **b** Amperometric response obtained for 7 different biosensors with 10 ng/ml of CEA, **c** Cathodic current obtained from ^CEA^Ab1-bound electrode during CV at 50 mV/s for 100 cycles in PBS containing HQ

To examine the specificity of the proposed electrochemical sensor, a series of experiments was performed with competing analytes like metabolite (uric acid [UA]), proteins (BSA, FBS), amino acids (l-arginine [l-Arg] and l-cysteine [l-Cyst]), vitamin (ascorbic acid [AA]) and cancer biomarker (alphafetoprotein [AFP]) (Fig. S9). From the histogram, it can be noted that the current response for interfering species was significantly less, whereas in case of CEA the response was significantly high demonstrating the high specificity of proposed biosensor towards CEA (Fig. 8a). Reproducibility of the biosensor was examined by measuring the response for 10 ng/ml CEA in seven different sensor electrodes using CUCDs@^CEA^Ab2 probe (Fig. S10). It was observed that all the seven electrodes afforded consistent response with a relative standard deviation (RSD %) of 7.3%, signifying good reproducibility of the sensor (Fig. 8b). The stability of ^CEA^Ab1 bound PEI-*g*-PAANI modified SPE was evaluated by conducting cyclic voltammetry for 100 cycles in PBS containing 3 mM HQ. CV response observed was consistent and showed 7.4% decrease in cathodic peak current even after 100th cycle demonstrating good stability of biosensor (Fig. 8c). Furthermore, CV response of antibody loaded sensor electrode was recorded for 20 days in 5 days interval. Electrode showed only 26% decrease in current after 20th day which showed the reasonable stability of sensor electrode (Fig. S11).

#### Estimation of CEA in human blood serum and spike-recovery analysis

To validate the sensor for practical analysis, the estimation of CEA in human blood serum sample was carried out (Fig. S12). The concentration of CEA in human serum sample was found to be 1.47 ng/ml which is consistent with the value obtained with the CMIA method. The RSD % between the values obtained from these two strategies were found to be 0.96% as shown in Table S4.

Proposed sensor was also assessed by spike-recovery analysis in which a known concentration of antigen standard (10, 20, 30 ng/ml of CEA) was spiked in the undiluted real human serum sample and amperometry measurement was carried out (Fig. S13). The result showed that the recovery was 95.0%, 106.2% and 96.3% (Table S5) for CEA respectively, indicating its potential for application in clinical analysis.

#### Viability of hDPSCs and MG63 cells against CUCDs

**Fig. 9:**
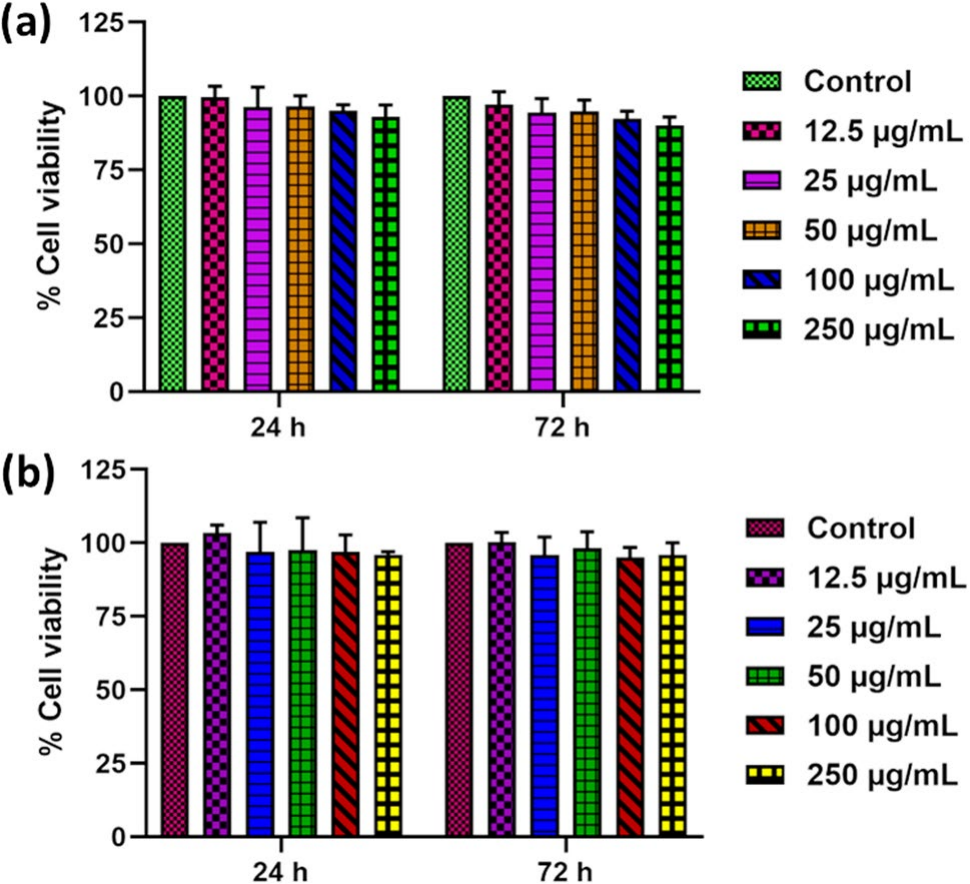
% cell viability of **a** human dental pulp stem cells (hDPSCs) and **b** MG63 osteosarcoma cells with different concentration of CUCDs for a duration of 24 and 72 h

From Fig. 9 it is seen that CUCDs are not toxic (cell viability > 90%) towards both the cell lines tested, at 24 and 72 h, indicating that it may not cause any adverse reaction when placed inside the human body or placed in contact with the skin. Human dental pulp stem cells are tall, columnar cells which differentiate from primitive mesenchymal cells. So, this is a good model for testing toxicity of materials. In addition, MG63 cells are widely used as an in vitro model of bone cancer to study the mechanisms underlying tumour development and to test the efficacy of potential therapeutic agents.

## Conclusion

In summary, an electrochemical sandwich immunosensor was designed over amine enriched PEI-*g*-PAANI modified SPE surface to quantify CEA using bio-functionalized CUCDs as detection probe. CUCDs based detection probe helped to enhance sensitivity of the immunosensor by 3.5 times as compared to conventional electrochemical immunosensor. Sensor also afforded low detection limit, wide linear range, high specificity without any interference signal from potential interferants. Designed immunosensor was used to detect CEA in human blood serum sample and measured values are consistent with the values obtained by conventional CMIA method. CUCDs used in the sensor was synthesized by hydrothermal treatment of cow urine. Presence of amino acids, urea, and nitrogen containing biomolecules in cow urine afforded inherent nitrogen containing functional groups to CUCDs which improved the fluorescence property and facilitated effective conjugation of antibody. In addition, cytotoxicity assessment by MTT assay revealed that CUCDs did not produce significant adverse effect on cells (hDPSCs and MG63 osteosarcoma cells), indicating good biocompatibility of CUCDs. The results of proposed immunosensor using CUCDs based detection probe and amine enriched sensing platform, reflect its significant potential in clinical application for the detection of critical cancer biomarkers towards early diagnosis of cancer.

### Supplementary Information

Additional file1 (DOCX 850 kb)

## Data Availability

All data generated or analyzed during this study are included in this published article (and its supplementary information files).

## Code availability

All source code generated or analyzed during this study are included in this published article (and its supplementary information files).
