# Supplementary material for: Bio-functionalized carbon dots for signaling immuno-reaction of carcinoembryonic antigen in an electrochemical biosensor for cancer biomarker detection
Source: Discov Nano. 2024 Feb 29;19(1):37. doi: 10.1186/s11671-024-03980-3 (PMC10904696; doi:10.1186/s11671-024-03980-3)
Supplement: Supplementary file 1 — Additional file1 (DOCX 850 kb) [file 11671_2024_3980_MOESM1_ESM.docx]

**Bio-functionalized carbon dots for signaling immuno-reaction of carcinoembryonic antigen in an electrochemical biosensor for cancer biomarker detection**

Amarnath Chellachamy Anbalagan^a^, Jyoti Korram^a^, Mukesh Doble^b^, and

Shilpa N. Sawant ^a, c,^ *

*^a^ Chemistry Division, Bhabha Atomic Research Centre, Trombay, Mumbai 400085, India.*

*^b^ Department of Cariology, Saveetha Dental College & Hospitals, Saveetha Institute of Medical and Technical Sciences, Chennai-600077, India.*

*^c^ Homi Bhabha National Institute, Anushaktinagar, Mumbai 400094, India.*

Correspondence: Telephone: +91 22 25593869, Email: stawde@barc.gov.in (Dr. Shilpa N. Sawant)

**Quantum yield (QY) measurement**

The quantum yield (QY) of CUCDs was obtained using equation:

$$Q=Q_{R}\frac{Y}{Y_{R}}\frac{I_{R}}{I}\frac{\eta^{2}}{\eta_{R}^{2}}$$

Where Q is the QY, Y is the optical density, I is the measured integrated emission intensity, and η is the refractive index of the solvent. The subscript “R” refers to the reference with known QY. For these aqueous solutions, η/η_R_=1. Quinine sulfate (0.1 M, H_2_SO_4_ as solvent; QY= 0.54) were chosen as standards (**Fig S1**). The relative quantum yield of CUCDs was found to be 5.69 % (**Fig S1**).

**
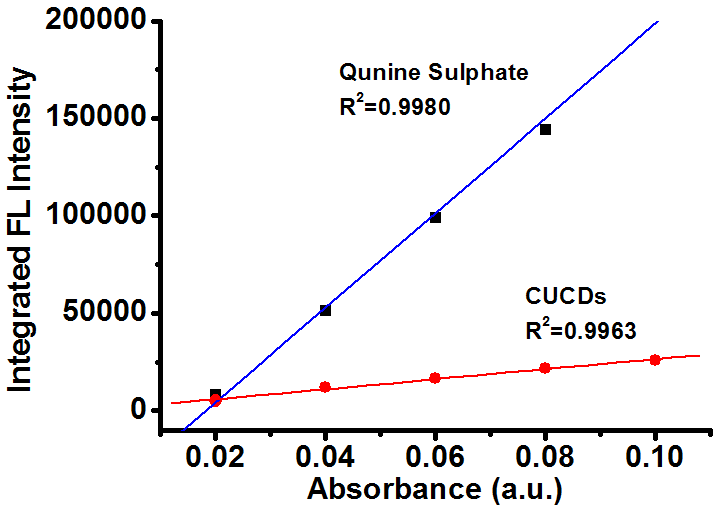
**

**Fig S1**: Calibration curve between absorbance (360 nm) and FL intensity (440 nm) for the determination of relative quantum yield of CUCDs.

**Table S1**: Quantum yield of various biomass derived carbon dots

| **SI. No.** | **Biomass source** | **Quantum yield (%)** | **Reference** |
| --- | --- | --- | --- |
| 1 | Bagasse | 4.7 | [1] |
| 2 | Soy milk | 2.6 | [2] |
| 3 | Cucumber juice | 3.25 | [3] |
| 4 | Citrum pectin | 1.1 | [4] |
| 5 | Carrot | 5.16 | [5] |
| 6 | Sugarcane juice | 5.67 | [6] |
| 7 | Starch | 21.7 | [7] |
| 8 | Banana petioles | 8.53 | [8] |
| 9 | Corn flour | 7.7 | [9] |

**Reproducibility of synthesis method**

Reproducibility is one of the general issues with the biomass derived nanomaterials. However, using biomass waste as starting material has several advantages including valorization of waste into value added products. To evaluate the reproducibility of CUCDs synthesis method, a set of CDs has been synthesized using urine samples from different cows (CUCDs_2_, CUCDs_3_, CUCDs_4_) and different urine samples from same cow collected at different time (CUCDs_mor_, CUCDs_noo_, CUCDs_nig_). The FL spectra was recorded for the synthesized CUCDs at excitation wave length 380 nm as depicted in **Fig S2**. Fluorescence spectra of CUCDs_mor_, CUCDs_noo_, CUCDs_nig_ showed emission at 449.5, 449.4 450.6 nm respectively which is close to the value of CUCDs emission values (450.3 nm) (**Fig S2**). FL spectra of CUCDs_2_, CUCDs_3_, CUCDs_4_ showed emission at 455.2, 453.5 and 451.3 nm (**Fig S2**). It was observed that all the seven samples showed nearly consistent emission values with a relative standard deviation (RSD %) of 0.5 %, signifying good reproducibility of the synthesis method.

**Fig S2**: FL spectra of CUCDs synthesized from cow urine collected from same cow at different times (CUCDs, early morning-CUCDs_mor_, noon-CUCDs_noo_, night-CUCDs_nig_) and cow urine collected from different cows (CUCDs_2_, CUCDs_3_, CUCDs_4_)

**Fig S3**: Electro-deposition of o-PDA (0.1 M) in 1 M HCl using CV at 50 mV/s.


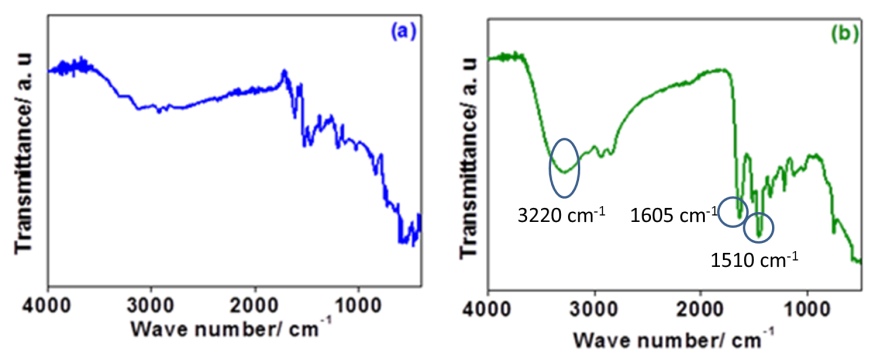


(c)

**Fig S4**: FTIR spectra of (a) PAANI, PEI-g-PAANI synthesized by (b) chemical method and (c) electrochemical pathway.

The FTIR spectra of glutaraldehyde and PEI are available in literature reports [10, 11]

**Fig S5**: (a) CV and Nyquist plot of (b) PEI-g-PAANI and (c) PANI before (x) and after bio-conjugation (y) in 1:1 mixture of 5 mM K_3­_[Fe(CN)_6_] and K_4­_[Fe(CN)_6_] containing 0.1 M KCl (inset: fitted circuit)

**Table S2**: Fitted parameters for equivalent circuit of unmodified and bio-functionalized PEI-g-PANI and Conventional PANI modified SPE

|  | PANI | PANI-^CEA^Ab1 | PEI-*g*-PAANI | PEI-*g*-PAANI- ^CEA^Ab1 |
| --- | --- | --- | --- | --- |
| R_E_/ Ω | 120.1 | 121.7 | 203 | 205.5 |
| R_CT_/ Ω | 15.8 | 35.8 | 50.5 | 255.5 |

ΔR_CT_ (PEI-*g*-PAANI- ^CEA^Ab1- PEI-*g*-PAANI) = 250 Ω; ΔR_CT_ (PANI- ^CEA^Ab1- PANI) = 20 Ω


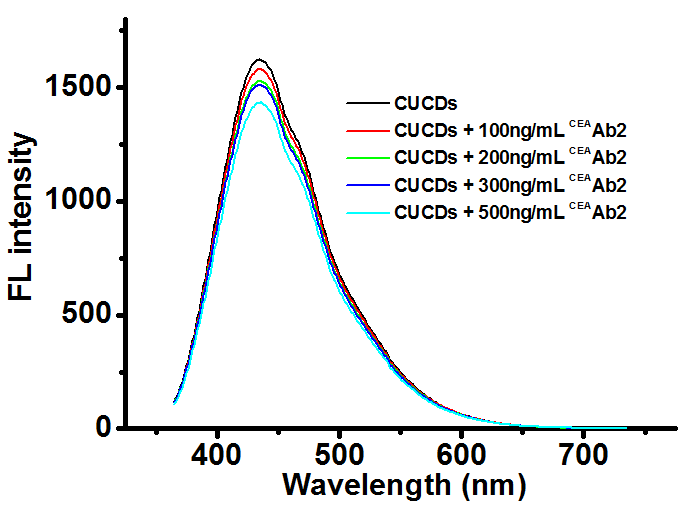


**Fig S6:** FL spectra of CUCDs with increasing concentration of antibody.

**Sensor constructed with CUCDs@^CEA^Ab2 probe**

**Fig S7**: Amperometric response of sensor in different concentration of CEA and CUCDs@^CEA^Ab2 detection probe in PBS containing 3 mM HQ and 3 mM H_2_O_2_.

**Sensor constructed with ^CEA^Ab2 probe**

**Fig S8**: Amperometric response of sensor in different concentration of CEA and ^CEA^Ab2 detection probe in PBS containing 3 mM HQ and 3 mM H_2_O_2_.

**Table S3**: Comparison of analytical performances of electrochemical immunosensors for CEA detection

| SI. No | Matrix | Detection probe | LOD | Linear range | Ref |
| --- | --- | --- | --- | --- | --- |
| 1 | polypyrrole (PPY) nanocomposite | NA | 33 pg/ml | 0.1-1000 ng/ml | [12] |
| 2 | Poly(3,4-ethylenedioxythiophene) (PEDOT) /polypyrrole films | NA | 80 pg/ml | 0.1-100000 ng/ml | [13] |
| 3 | graphene/PEDOT | NA | 450 pg/ml | 0.77–14 ng/ml | [14] |
| 4 | Au | Lecitin-HRP | 3.4 pg/ml | 5-40 ng/mL | [15] |
| 5 | graphene oxide/Au NPs /poly(indole-6-carboxylic acid) | NA | 20 ng/ml | 0.02-90 ng/ml | [16] |
| 6 | Lipoic acid N-hydroxysuccinimide ester | Au-Ferrocene | 10 pg/ml | 0.05-20 ng/ml | [17] |
| 7 | gold nanoparticles/prussian blue-poly(3,4-ethylenedioxythiophene) nanocomposite | NA | 10 pg/ml | 0.05-40 ng/ml | [18] |
| 8 | Hemin-rGO-AuNPs | HRP-Cu_3_(PO_4_)_2_  -HNF-AuNPs) | 29 fg/ml | 100 fg/ml - 100 ng ml | [19] |
| 9 | GCE-Au NPs | MoS_2_-g-C_3_N_4_-PtCu | 33 fg/ml | 100 fg/ml to 80 ng/ml | [20] |
| 10 | Au NPs | Cu_2_O@Cu-MOF@Au | 17 fg/ml | 50 fg/ml – 80 ng/ml | [21] |

**Interference study**

**Fig S9**: Amperometric response of sensors towards potential interferents like BSA, FBS, ascorbic acid, uric acid, L-cysteine, L-arginine and AFP (50 ng/ml)

**Reproducibility study**

**Fig S10**: Amperometric response of different sensors constructed with 10 ng/ml CEA

**Stability Study**

**Fig S11**: Cathodic current response from ^CEA^Ab1-bound electrode over a period of 20 days.

**Analysis of real blood serum samples**

**Fig S12**: Amperometric response of human blood serum sample using proposed biosensor

**Table S4**: CEA concentration in human blood serum samples determined by proposed sensor and conventional CMIA

| Serum sample | [CEA]^#^ in serum (ng/ml) | [CEA] in serum by CMIA (ng/ml) | RSD (%) |
| --- | --- | --- | --- |
| A | 1.47 | 1.45 | 0.96 |

^#^ detected by proposed sensor

**Spike-recovery**

**Fig S13:** Amperometric response of antigen spiked human blood serum samples

**Table S5**: Spike-recovery of CEA in human blood serum sample

| [CEA]^#^ in serum (ng/ml) | [CEA] in serum by CMIA (ng/ml) | [CEA] spiked (ng/ml) | Total [CEA] detected (ng/ml) | Recovery of CEA (%) |
| --- | --- | --- | --- | --- |
| 1.47 | 1.45 | 10 | 10.9 | 95.0 |
| 1.47 | 1.45 | 20 | 22.8 | 106.2 |
| 1.47 | 1.45 | 30 | 30.3 | 96.3 |

^#^ detected by proposed sensor

**References**

1. Liu XJ, Guo ML, Huang J, Yin XY (2013) Improved Fluorescence of Carbon Dots Prepared from Bagasse under Alkaline Hydrothermal Conditions. BioResources. 8: 2537. 10.15376/biores.8.2.2537-2546
2. Zhu C, Zhai J, Dong S (2012) Bifunctional fluorescent carbon nanodots: green synthesis via soy milk and application as metal-free electrocatalysts for oxygen reduction. Chem Commun 48:9367. 10.1039/C2CC33844K
3. Wang C, Sun D, Zhuo K, Zhang H, Wang J (2014) Simple and green synthesis of nitrogen-, sulfur-, and phosphorus-co-doped carbon dots with tunable luminescence properties and sensing application. RSC advances 4:54060. <https://doi.org/10.1039/C4RA10885J>
4. Zhao XJ, Zhang WL, Zhou ZQ (2014) Sodium hydroxide-mediated hydrogel of citrus pectin for preparation of fluorescent carbon dots for bioimaging. Colloids Surf. B: Biointerfaces 123:493. <https://doi.org/10.1016/j.colsurfb.2014.09.048>
5. Liu Y, Liu Y, Park M, Park SJ, Zhang Y, Akanda MR, Park BY, Kim HY (2017) Green synthesis of fluorescent carbon dots from carrot juice for in vitro cellular imaging. Carbon Lett 28;21:61. http://dx.doi.org/10.5714/CL.2017.21.061
6. Mehta VN, Jha S, Kailasa SK (2014) One-pot green synthesis of carbon dots by using Saccharum officinarum juice for fluorescent imaging of bacteria (Escherichia coli) and yeast (Saccharomyces cerevisiae) cells. Mater Sci Eng C 38:20. <https://doi.org/10.1016/j.msec.2014.01.038>
7. Li W, Liu Y, Wu M, Feng X, Redfern SA, Shang Y, Yong X, Feng T, Wu K, Liu Z, Li B (2018) Carbon‐quantum‐dots‐loaded ruthenium nanoparticles as an efficient electrocatalyst for hydrogen production in alkaline media. Adv Mater 30:1800676. <https://doi.org/10.1002/adma.201800676>
8. Korram J, Koyande P, Mehetre S, Sawant SN (2023) Biomass-Derived Carbon Dots as Nanoprobes for Smartphone–Paper-Based Assay of Iron and Bioimaging Application. ACS omega 8:31410. <https://doi.org/10.1021/acsomega.3c03969>
9. Wei J, Zhang X, Sheng Y, Shen J, Huang P, Guo S, Pan J, Feng B (2014) Dual functional carbon dots derived from cornflour via a simple one-pot hydrothermal route. Mater Lett 123:107. <https://doi.org/10.1016/j.matlet.2014.02.090>
10. Patro TU, Wagner HD (2016) Influence of graphene oxide incorporation and chemical cross-linking on structure and mechanical properties of layer-by-layer assembled poly(Vinyl alcohol)-Laponite free-standing films. J Polym Sci Part B Polym Phys 54:2377. https://doi.org/10.1002/polb.24226
11. Wei Y, Salih KAM, Hamza MF, Fujita T, Rodríguez-Castellón E, Guibal E (2021) Synthesis of a new phosphonate-based sorbent and characterization of its interactions with lanthanum (III) and terbium (III). Polymers 13:1513. https://doi.org/10.3390/polym13091513
12. Song J, Teng H, Xu Z, Liu N, Xu L, Liu L, Gao F, Luo X (2021) Free-standing electrochemical biosensor for carcinoembryonic antigen detection based on highly stable and flexible conducting polypyrrole nanocomposite. Microchim Acta 188:217. <https://doi.org/10.1007/s00604-021-04859-1>
13. Carneiro LPT, Ferreira NS, Tavares APM, Pinto AMFR, Mendes A, Sales MGF (2021) A passive direct methanol fuel cell as transducer of an electrochemical sensor, applied to the detection of carcinoembryonic antigen. Biosens Bioelectron 175:112877. <https://doi.org/10.1016/j.bios.2020.112877>
14. Yen YK, Chao CH, Yeh YS (2020) A graphene-PEDOT:PSS modified paper-based aptasensor for electrochemical impedance spectroscopy detection of tumor marker. Sensors 20:1372. 10.3390/s20051372
15. Wang QL, Cui HF, Song X, Fan SF, Chen LL, Li MM, Li ZY (2018) A label-free and lectin-based sandwich aptasensor for detection of carcinoembryonic antigen. Sens Actuators B: Chem 260:48. <https://doi.org/10.1016/j.snb.2017.12.105>
16. Zhao D, Wang Y, Nie G (2016) Electrochemical immunosensor for the carcinoembryonic antigen based on a nanocomposite consisting of reduced graphene oxide, gold nanoparticles and poly(indole-6-carboxylic acid). Microchim Acta 183:2925. <https://doi.org/10.1007/s00604-016-1940-2>
17. Gu X, She Z, Ma T, Tian S, Kraatz HB (2018) Electrochemical detection of carcinoembryonic antigen. Biosens Bioelectron 102:610.
18. Yang T, Gao Y, Liu Z, Xu J, Lu L, Yu Y (2017) Three-dimensional gold nanoparticles/prussian blue-poly(3,4-ethylenedioxythiophene) nanocomposite as novel redox matrix for label-free electrochemical immunoassay of carcinoembryonic antigen, Sens Actuators B: Chem 239:76. <https://doi.org/10.1016/j.snb.2016.08.001>
19. Xu L, Liu Z, Lei S, Huang D, Zou L, Ye B (2019) A sandwich-type electrochemical aptasensor for the carcinoembryonic antigen via biocatalytic precipitation amplification and by using gold nanoparticle composites. Microchim Acta 186:473.

<https://doi.org/10.1007/s00604-019-3542-2>

1. Song Y, Qiao J, Li W, Ma C, Chen S, Li H, Hong C (2020) Bimetallic PtCu nanoparticles supported on molybdenum disulfide–functionalized graphitic carbon nitride for the detection of carcinoembryonic antigen. Microchim. Acta 187:538. <https://doi.org/10.1007/s00604-020-04498-y>
2. Li W, Yang Y, Ma C, Song Y, Hong C, Qiao X (2020) A sandwich-type electrochemical immunosensor for ultrasensitive detection of CEA based on core–shell Cu_2_O@Cu-MOF@Au NPs nanostructure attached with HRP for triple signal amplification, J Mater Sci 55:13980. <https://doi.org/10.1007/s10853-020-04904-z>
